# Kondo-like zero-bias conductance anomaly in a three-dimensional topological insulator nanowire

**DOI:** 10.1038/srep21767

**Published:** 2016-02-25

**Authors:** Sungjae Cho, Ruidan Zhong, John A. Schneeloch, Genda Gu, Nadya Mason

**Affiliations:** 1 Department of Physics, Korea Advanced Institute of Science and Technology, Daejeon 305-701, Republic of Korea; 2 Condensed Matter Physics and Materials Science Department, Brookhaven National Laboratory, Upton, NY 11973, USA; 3 Department of Physics and Frederick Seitz Materials Research Laboratory, University of Illinois, 104 South Goodwin Avenue, Urbana, Illinois 61801, USA

## Abstract

Zero-bias anomalies in topological nanowires have recently captured significant attention, as they are possible signatures of Majorana modes. Yet there are many other possible origins of zero-bias peaks in nanowires—for example, weak localization, Andreev bound states, or the Kondo effect. Here, we discuss observations of differential-conductance peaks at zero-bias voltage in non-superconducting electronic transport through a 3D topological insulator (Bi_1.33_Sb_0.67_)Se_3_ nanowire. The zero-bias conductance peaks show logarithmic temperature dependence and often linear splitting with magnetic fields, both of which are signatures of the Kondo effect in quantum dots. We characterize the zero-bias peaks and discuss their origin.

The three dimensional topological insulator (3D TI) is a new class of material having metallic surface states inside a bulk band gap^1^,^2^,^3^. The topological surface states are characterized by gapless Dirac dispersions and novel properties such as momentum-spin locking, which were confirmed by angle-resolved photoemission spectroscopy (ARPES)^4^,^5^,^6^, scanning tunneling spectroscopy (STS)^7^,^8^,^9^,^10^,^11^ and electrical transport measurements^12^,^13^,^14^,^15^,^16^,^17^,^18^,^19^. 3D TI nanowires with an insulating bulk, which can be described as a hollow metallic cylinder, have shown Aharonov-Bohm oscillations when a magnetic flux is threaded through the axis^20^,^21^ and Coulomb blockade behavior when connected to metal electrodes through ultrathin TI tunnel barriers^22^. Recently, TI nanowires in the proximity of s-wave superconductors have been predicted to harbor Majorana bound states^23^,^24^, a transport signature of which is a zero-bias tunneling-conductance peak. Similar proximity-coupled topological nanowire systems of InSb^25^ and Fe^26^ have recently demonstrated Majorana-like zero-bias anomalies, leading to a worldwide effort to better understand the origin and behavior of zero-bias anomalies in topological wires. Zero-bias conductance peaks, not observed yet in 3D TI nanowires, are novel features which may be related to various physical origins such as weak antilocalization, Andreev bound states, and Kondo effect besides Majorana bound states^27^,^28^,^29^. Here we report the first observation of zero-bias anomalies in non-superconducting electronic transport through a 3D TI nanowire contacted by two metal electrodes. We also observed a logarithmic temperature dependence and linear splitting with magnetic fields, both of which imply that the zero-bias peaks result from Kondo-like effect.

## Results

**Figure 1:**
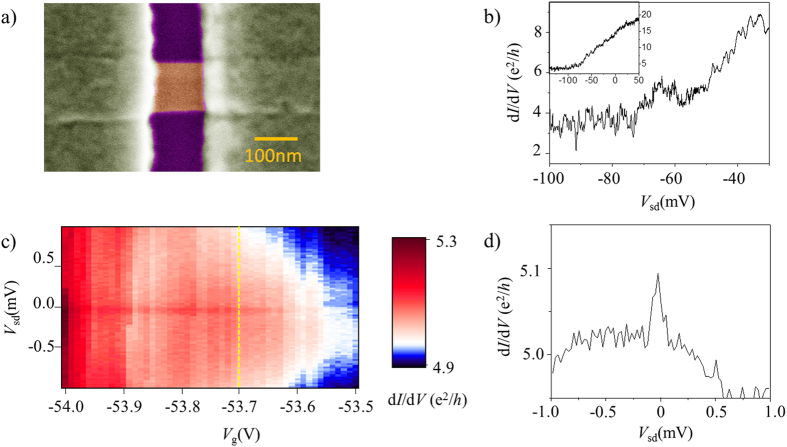
Device image and characterization. **(a)** False-colored Scanning Electron Microscope image of the two-terminal topological insulator nanowire device. **(b)** Two-probe differential conductance d*I*/d*V* as a function of back-gate voltage *V*_g_ at *B* = 50 mT and *T* = 16 mK at zero bias-voltage. (**c**,**d**) Two-dimensional plot of G(*V*_sd_,*V*_g_) and G(*V*_sd_) at *V*_g_ = −53.7 V measured at perpendicular magnetic field B = 200 mT.

We have grown bulk (Bi_1.33_Sb_0.67_)Se_3_ crystals and confirmed the existence of the surface states inside the bulk band gap by ARPES, as described elsewhere^30^. Using the “scotch tape method”, we obtained naturally cleaved topological insulator nanowires on 300 nm SiO_2_/highly *n-*doped Si substrates. Subsequently, we characterized and chose nanowires having widths ≤100 nm and thickness >12 nm by atomic force microscope, to avoid unwanted wavefunction hybridizations of top and bottom surfaces^31^. Widths of nanowires were measured again, after all the electrical measurements were done, by scanning electron microscopy (SEM). Immediately after we chose nanowires and identified their locations on a SiO_2_/Si chip, we spun an electron-beam resist (Microchem Corp. PMMA 950 A4) double layer at 4000 rpm to avoid possible contamination or excessive doping of the nanowire surfaces by long exposure to air^32^. Subsequently, we performed electron beam lithography, developed to remove the resist in the regions of source/drain electrodes, and finally deposited Ti/Al (2.5 nm /150 nm) with an Au 10 nm capping following a brief ion milling at low power. The devices were wire-bonded and cooled-down in a commercial dilution refrigerator immediately following lift-off in acetone for 1 hour. Figure 1(a) is a false-colored SEM image of a completed device, where a small section of the nanowire having dimensions of width ~90 nm, length ~90 nm, and thickness ~13 nm is contacted by a source and a drain electrode.

Here, we report our two-probe differential conductance measurements made between Ti/Al source-drain electrodes driven normal (non-superconducting). To do this, we applied small perpendicular magnetic fields of 30 − 200 mT, which is above the critical field of the Al electrodes ~12 mT (see Supplementary Info.). Figure 1(b) shows that as gate voltage decreased, differential conductance (d*I*/d*V*) at zero-bias voltage decreased due to the decrease in the density of states, and finally as gate voltages exceeded *V*_g_ = −80 V, the conductance reached its minimum implying that the chemical potential is tuned near the charge neutrality point and therefore a minimum density of states. Due to the low electron doping of our Sb-doped crystals and exfoliated devices as reported previously^21^,^30^, we are able to tune the chemical potential effectively below the bottom of the bulk conduction band using a back-gate through SiO_2_ in our nanowire device. In all range of gate voltages, we observed reproducible conductance fluctuations reminiscent of Coulomb charging effect or phase coherent interference effects such as Fabry-Perot and universal conductance fluctuations.

**Figure 2:**
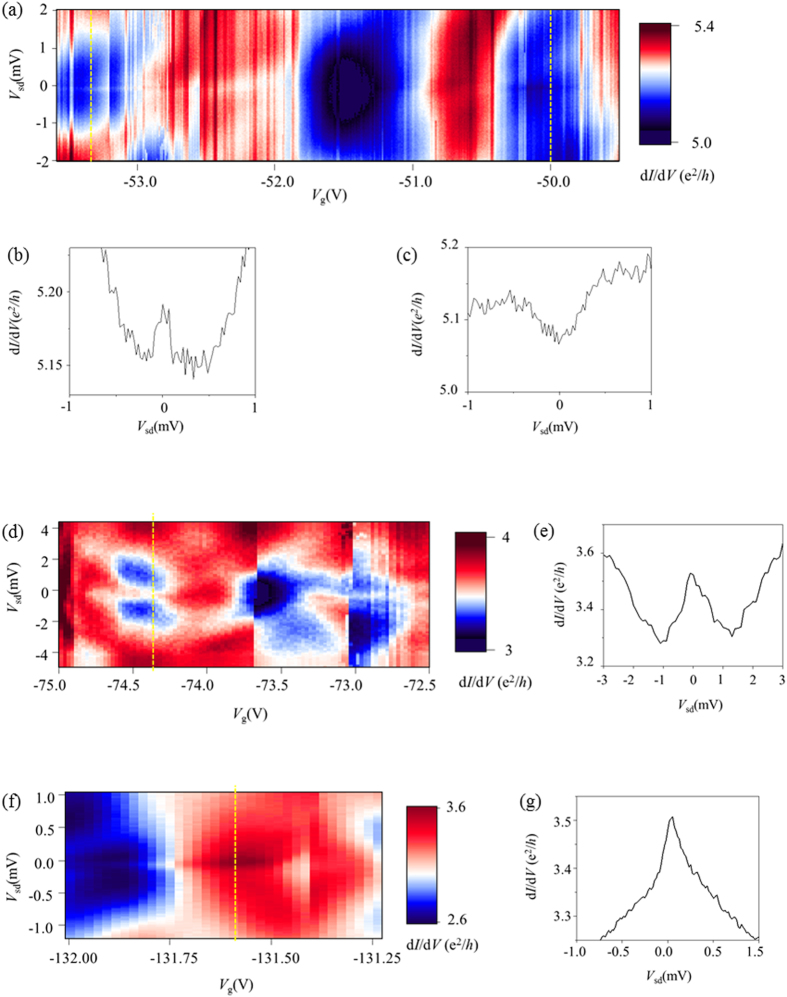
Differential conductance spectroscopy. (**a**,**d**,**f)** Two-dimensional plots of G(*V*_sd_,*V*_g_) measured with perpendicular magnetic field B = 200 mT applied in different gate voltage ranges. **(b**,**c**,**e**,**g)** Differential conductance as a function of bias voltage at fixed gate voltages, **(b)**−53.3 V, **(c)**−53.0 V, **(e)**−74.4 V, and **(g)**−131.6 V. Each gate voltage whose differential conductance was plotted is marked with yellow dotted-lines in the two-dimensional plots **(a**,**d**,**f)**.

Figure 1(c) shows differential conductance spectroscopy, a two-dimensional plot of G(*V*_sd_,*V*_g_) measured while varying dc source-drain bias-voltages *V*_sd_ at different gate voltages *V*_g._ The transport spectroscopy unexpectedly showed enhanced zero-bias conductance persisting with gate voltages at *V*_g_ < −50 V where the background conductance ≤ 5 e^2^/*h*. Figure 1(d) shows differential conductance as a function of bias voltages at *V*_g_ = −53.7 V. We have further performed differential conductance spectroscopy measurements in wider ranges of gate voltages as shown in Fig. 2. Zero-bias conductance peaks having amplitudes ranging from 0.05 to 0.7 e^2^/*h* were observed in a wide range of gate voltages. Often, zero-bias conductance dips appeared in certain regions of gate voltages as shown in Fig. 2(a,c). In the rest of this letter, we discuss the possible origin of the observed zero-bias conductance anomaly.

## Discussions

Recently, zero-bias peaks observed in 1D topological superconductors, i.e., a strong spin-orbit coupled semiconducting nanowires in the proximity of superconductors under a parallel magnetic field, captured significant attention. The zero-bias anomaly in those systems have been explained as a signature of Majorana zero modes^23^. There have been similar theoretical proposals on producing Majorana zero modes in topological insulator (Bi_2_Se_3_) nanowires proximity-coupled to s-wave superconductors under a parallel magnetic field^23^,^24^. However, the absence of superconductivity in a magnetic field higher than the critical field of the Al electrodes in our setup excludes the Majorana zero modes among possible explanations for our zero-bias peaks. Due to the lack of superconductivity, Andreev states bound to superconductors also do not provide an explanation for our observed zero-bias conductance peaks.

**Figure 3:**
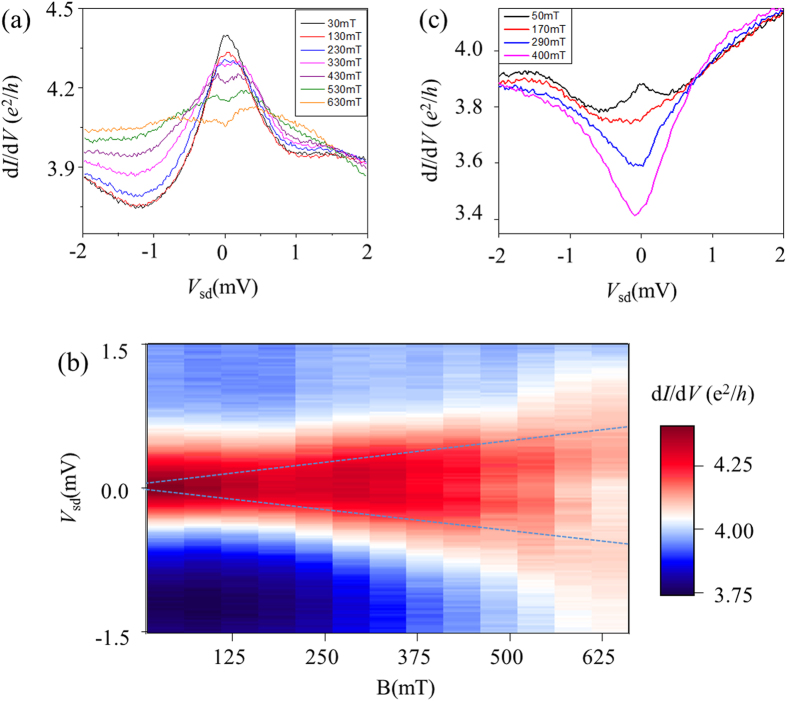
Evolution of zero-bias conductance peaks with magnetic fields. (**a**) Differential conductance as a function of bias voltages at a fixed gate *V*_g_ = −69 V and different magnetic fields. (**b**) Two-dimensional plots of **(a)**. Blue lines show splitting of the zero-bias conductance peaks with magnetic fields. **(c)** Differential conductance as a function of bias voltages at a fixed gate *V*_g_ = −65 V and different magnetic fields.

To understand the origin of the zero-bias conductance peaks, we have performed the transport measurements at different magnetic fields. Figure 3 shows two different ways in which peaks evolve with magnetic fields at fixed gate voltages (see Supplementary Fig. S4 for 2D G(*V*_sd_,*V*_g_) plot). Peaks having large amplitude (>0.4 e^2^/h, such as the one) measured at *V*_g_ = −69 V, split with magnetic fields (Fig. 3(a,b)). The formation of zero-bias peaks and the splitting of the peaks with a magnetic field are reminiscent of the Kondo effect in quantum dots. A characteristic feature of the Kondo effect in quantum dots is that the zero-bias peak splits with magnetic field at 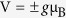 *B* (the Zeeman splitting), where μ_B_ is the Bohr magneton. Assuming the relation of the zero-bias peaks to Kondo effect, we estimate the Zeeman g-factor of the surface states in our topological insulator (Bi_1.33_Sb_0.67_)Se_3_ nanowire is ~15 from the blue line in Fig. 3(b), which is roughly half the reported g factor value of the bulk states in Bi_2_Se_3_^33^. On the other hand, peaks of relatively small amplitude (<0.1 e^2^/*h*), such as that observed at *V*_g_ = −65 V, switched to conductance dips without splitting as the magnetic field was increased (Fig. 3(c)). This collapse of the zero-bias peaks having small peak amplitudes (<0.1 e^2^/h) with a magnetic field, without being splitted, and having large background conductance is most likely due to lower Kondo temperatures (*T* ~ *T*_K_) at these gate voltages^34^.

**Figure 4:**
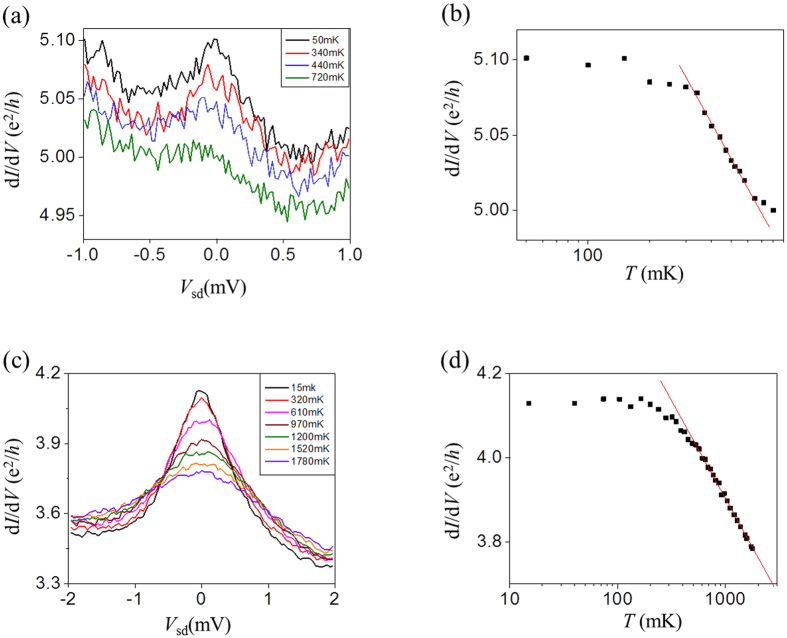
Temperature dependence of zero-bias conductance peaks. Temperature dependence of zero-bias conductance peaks observed at **(a**,**b)** *V_g_* = −60 V, and **(c**,**d**) *V_g_* = −72.7 V. Conductance of peaks having both small and large amplitudes decreased logarithmically with temperature.

To further investigate the observed Kondo-like anomaly at zero-bias voltage, we measured temperature dependence of the zero-bias peaks. Figure 4 shows that conductance of peaks having both small and large amplitudes increases logarithmically with decreasing temperature and eventually saturated to a constant conductance value as *T* → 0^35^ (see also Supplementary Fig. S4 for 2D G(*V*_sd_,*V*_g_) plot). In the high-temperature limit, Small peaks with large background conductance vanished at relatively low temperatures <1 K as shown in Fig 4(a), and the large peaks with relatively small conductance background did not vanish up to the temperature limit (~1.8 K) in our dilution refrigerator. Both the logarithmic temperature dependence and the peak splitting with magnetic field suggest that the zero-bias peaks are Kondo-like effects in a quantum dot. We obtain rough estimates of the Kondo temperatures, *T*_K,_ which ranges from 300 mK to 5 K, by equating the full-width at half-maximum of different zero-bias conductance peaks at the base temperature to 2*k*_B_*T*_K_/e^35^,^36^.

The Kondo effect describes the upturn of the resistance of metals at low temperatures when magnetic impurities are added^37^. A similar Kondo effect in semiconducting nanostructures results from a bound state formed between a local spin in a quantum dot and the electrons in the reservoir of source/drain electrodes. We find that the overall background conductance values in our experiments are relatively high (3*e*^2^/*h* < *G* < 5*e*^2^/*h*) compared to the conductance values (*G* < 2*e*^2^/*h*) previously reported in quantum dots showing the Kondo effect^35^,^36^, and that our topological insulator nanowire device behaves as an open quantum dot. Coulomb charging is required in order for the Kondo effect to be observed in quantum dots; this is usually observed in a system having low dot-electrode transmission probabilities and conductance less than 2e^2^/*h*. However, Coulomb charging effects have often been observed in open quantum dots where 2 e^2^/*h* < *G* < 6 e^2^/*h* 38,39,40. Therefore, high overall conductance values of 3e^2^/*h* < *G* < 5e^2^/*h* does not necessarily exclude the possibility of Coulomb charging and the Kondo effect in our open quantum dot device. The large, oval regions of low-conductance shown in the 2D transport spectroscopy G(*V*_sd_, *V*_g_) of Fig. 2(a) at −54 < *V*_g_ < −53, −52 < *V*_g_ < −51and −50.5 < *V*_g_ < −49.5 are consistent with a diamond-shaped Coulomb blockade in the presence of high lead-dot transparency. Although we do not clearly observe the even-odd parity behavior typical for the Kondo effect in quantum dots, several experiments of Kondo resonances have reported the absence of even-odd parity behavior^41^,^42^,^43^, due to either the formation of higher spin states (spin-triplet Kondo resonances) or correlation effects due to electron-electron interaction dominating over the confinement effect in quantum dots.

We often observe zero-bias peaks that increased with increasing magnetic field (see Supplementary Info.). This has previously been explained by singlet-triplet transitions of electron spin states in a quantum dot^27^. The magnetic-field-induced zero-bias peaks persist up to magnetic fields as large as *B* = 630 mT. We currently do not understand the physical mechanism of these magnetic-field-induced zero-bias peaks, although it is possible that they are related to the unique spin-momentum locking on the surface of topological insulators. In this case, it may be more energetically favorable to create a multi-particle triplet state than a single-particle spin-1/2 state. To our knowledge, no theoretical and experimental research has been reported on the subject and further studies are required to understand the physics of possible singlet-triplet transitions in topological insulator nanowires. Pikulin *et al.* pointed out in their simulation studies that weak antilocalization by disorder can also be a source of zero-bias conductance peaks at non-zero magnetic fields which break time-reversal symmetry^29^. However, this scenario is only possible when the 1D system has particle-hole symmetry resulting from superconductivity. Without the particle-hole symmetry, weak antilocalization effects should disappear when a magnetic field breaks time-reversal symmetry. The behavior of magnetic-field-induced zero-bias peaks persisting up to *B* = 630 mT in our device cannot be explained by a weak antilocalization effect, considering the absence of superconductivity. Moreover, lowering background conductance by increasing the tunnel barriers between electrodes and nanowires by a gate voltage is expected to suppress the zero-bias peaks originating from weak antilocalization. Our observation is opposite to this scenario: zero-bias peaks are absent in the gate voltage regions of *V*_g_ ≥ −40 V where conductance is higher, but more prominent as the gate voltage drops below −50 V and conductance decreases. This observation implies that weak antilocalization is not the origin of the zero-bias peaks in our device.

## Conclusion

In conclusion, we have observed zero-bias conductance peaks in non-superconducting transport through a topological insulator nanowire contacted by source-drain electrodes. The logarithmic temperature dependence and splitting of the peaks with magnetic fields strongly imply that the zero-bias peaks occur from Kondo-like origins in a quantum dot. Additional features differing from typical Kondo effects in quantum dots such as high background conductance (>2 e^2^/*h*) and absence of even-odd parity behavior were observed, which may be consistent with a singlet-triplet Kondo effect and related to the topological nature of the nanowires.

## Methods

### Device fabrication and measurement

Topological insulator nanowires were obtained by mechanical exfoliation (‘scotch tape method’) from bulk crystals of Bi_1.33_Sb_0.67_Se_3_, which were grown by a modified floating zone method^27^. After mechanical exfoliations of bulk crystals onto 300 nm SiO_2_/highly *n*-doped Si substrates, the nanowires were found under optical microscope. The dimensions of nanowires were measured by Atomic Force Microscopy and Scanning Electron Microscopy. Electron beam lithography and metal (Ti/Al/Au = 2.5 nm/150 nm/10 nm) deposition were used to pattern two-terminal devices on the nanowires. Completed devices were wire-bonded and cooled-down in a commercial dilution refrigerator (base temperature = 16 mK). The electrical measurements were performed using standard ac lock-in techniques.

## Additional Information

**How to cite this article**: Cho, S. *et al.* Kondo-like zero-bias conductance anomaly in a three-dimensional topological insulator nanowire. *Sci. Rep.* **6**, 21767; doi: 10.1038/srep21767 (2016).

## Supplementary Material

Supplementary Information

## References

[b1] Hasan M. Z. & Kane C. L. Colloquium: topological insulators. Rev. Mod. Phys. 82, 3045 (2010).

[b2] Fu L., Kane C. L. & Mele E. J. Topological insulators in three dimensions. Phys. Rev. Lett. 98, 106803 (2007). 17358555 10.1103/PhysRevLett.98.106803

[b3] Zhang H. J. *et al.* Topological insulators in Bi_2_Se_3_, Bi_2_Te_3_ and Sb_2_Te_3_ with a single Dirac cone on the surface. Nature Phys. 5, 438–442 (2009).

[b4] Hsieh D. *et al.* A tunable topological insulator in the spin helical Dirac transport regime. Nature 460, 1101–1105 (2009). 19620959 10.1038/nature08234

[b5] Chen Y. L. *et al.* Experimental realization of a three-dimensional topological insulator Bi_2_Te_3_. Science 325, 178–181 (2009). 19520912 10.1126/science.1173034

[b6] Xia Y. *et al.* Observation of a large-gap topological-insulator class with a single Dirac cone on the surface. Nature Phys. 5, 398–402 (2009).

[b7] Zhang T. *et al.* Experimental demonstration of topological surface states protected by time-reversal symmetry. Phys. Rev. Lett. 103, 266803 (2009). 20366330 10.1103/PhysRevLett.103.266803

[b8] Alpichshev Z. *et al.* STM imaging of electronic waves on the surface of Bi_2_Te_3_: Topologically protected surface states and hexagonal warping effects. Phys. Rev. Lett. 104, 016401 (2010). 20366373 10.1103/PhysRevLett.104.016401

[b9] Hanaguri T., Igarashi K., Kawamura M., Takagi H. & Sasagawa T. Momentum-resolved Landau-level spectroscopy of Dirac surface state in Bi_2_Se_3_. Phys. Rev. B 82, 081305R (2010).

[b10] Cheng P. *et al.* Landau quantization of topological surface states in Bi_2_Se_3_. Phys. Rev. Lett. 105, 076801 (2010). 20868065 10.1103/PhysRevLett.105.076801

[b11] Beidenkopf H. *et al.* Spatial fluctuations of helical Dirac fermions on the surface of topological insulators. Nature Phys. 7, 939–943 (2011).

[b12] Steinberg H., Gardner D. R., Lee Y. S. & Jarillo-Herrero P. Surface state transport and ambipolar electric field effect in Bi_2_Se_3_ nanodevices. Nano Lett. 10, 5032–5036 (2010). 21038914 10.1021/nl1032183

[b13] Qu D., Hor Y. S., Xiong J., Cava R. J. & Ong N. P. Quantum oscillations and Hall anomaly of surface states in the topological insulator Bi_2_Te_3_. Science 329, 821–824 (2010). 20671155 10.1126/science.1189792

[b14] Xu Y. *et al.* Observation of topological surface state quantum Hall effect in an intrinsic three-dimensional topological insulator. Nature Phys. 10, 956–963 (2014).

[b15] Analytis J. G. *et al.* Two-dimensional surface state in the quantum limit of a topological insulator. Nature Phys. 6, 960–964 (2010).

[b16] Kim D. *et al.* Surface conduction of topological Dirac electrons in bulk insulating Bi_2_Se_3_. Nature Phys. 8, 459–463 (2012).

[b17] Chen J. *et al.* Gate_voltage control of chemical potential and weak antilocalization in Bi_2_Se_3_. Phys. Rev. Lett. 105, 176602 (2010). 21231064 10.1103/PhysRevLett.105.176602

[b18] Checkelsky J. G., Hor Y. S., Cava R. J. & Ong N. P. Surface state conduction observed in voltage-tuned crystals of the topological insulator Bi_2_Se_3_. Phys. Rev. Lett. 106, 196801 (2010). 21668185 10.1103/PhysRevLett.106.196801

[b19] Yoshimi R. *et al.* Quantum Hall effect on top and bottom surface states of topological insulator (Bi_1−*x*_Sb_*x*_)_2_Te_3_ films. Nature Commun. 6, 6627 10.1038/ncomms7627 (2015). 25868494

[b20] Peng H. *et al.* Aharonov-Bohm interference in topological insulator nanoribbons. Nat. Mater. 9, 225–229 (2010). 20010826 10.1038/nmat2609

[b21] Cho S. *et al.* Aharonov–Bohm oscillations in a quasi-ballistic three-dimensional topological insulator nanowire. Nat. Commun. 6, 7634 10.1038/ncomms8634 (2015). 26158768

[b22] Cho S. *et al.* Topological Insulator Quantum Dot with Tunable Barriers. Nano Lett., 12, 469–472 (2012). 22181853 10.1021/nl203851g

[b23] Cook A. & Franz M. Majorana fermions in a topological-insulator nanowire proximity-coupled to an *s* -wave superconductor. Phys. Rev. B 84, 201105(R) (2011)

[b24] de Juan F., Ilan R. & Bardarson J. H. Robust Transport Signatures of Topological Superconductivity in Topological Insulator Nanowires, Phys. Rev. Lett. 113, 107003 (2014). 25238379 10.1103/PhysRevLett.113.107003

[b25] Mourik V. *et al.* Signatures of Majorana Fermions in Hybrid Superconductor-Semiconductor Nanowire Devices. Science 336, 1003–1007 (2012) 22499805 10.1126/science.1222360

[b26] Nadj-Perge S. *et al.* Observation of Majorana fermions in ferromagnetic atomic chains on a superconductor, Science 346, 602–607 (2014) 25278507 10.1126/science.1259327

[b27] Sasaki S. *et al.* Kondo effect in an integer-spin quantum dot. Nature 405, 764–767 (2000). 10866190 10.1038/35015509

[b28] Zareyan M., Belzig W., Nazarov, Yu & V. Superconducting proximity effect in clean ferromagnetic layers. Phys. Rev. B 65, 184505 (2002).

[b29] Pikulin D. *et al.* A zero-voltage conductance peak from weak antilocalization in a Majorana nanowire. New J. Phys. 14, 125011 (2012).

[b30] Cho S. *et al.* Symmetry protected Josephson supercurrents in three-dimensional topological insulators. Nat. Commun. 4, 1689 10.1038/ncomms2701 (2013). 23575693

[b31] Cho S., Butch N. P., Paglione J. & Fuhrer M. S. Insulating behavior in ultrathin bismuth selenide field effect transistors. Nano Lett. 11, 1925–1927 (2011). 21486055 10.1021/nl200017f

[b32] Kong D. *et al.* Rapid surface oxidation as a source of surface degradation factor for Bi_2_Se_3_. ACS Nano 5, 4698–4703 (2011). 21568290 10.1021/nn200556h

[b33] KÖhler H. & WÖchner E. The *g*-factor of the conduction electrons in Bi_2_Se_3_. Phys. Status Solidi B 67, 665 (1975).

[b34] Cronenwett S. M. *et al.* Low-temperature fate of the 0.7 structure in a point contact: a Kondo-like correlated state in an open system, Phys. Rev. Lett. 88, 226805 (2002). 12059445 10.1103/PhysRevLett.88.226805

[b35] Nygård J. *et al.* Kondo physics in carbon nanotubes, Nature 408, 342–346 (2000). 11099037 10.1038/35042545

[b36] van der Wiel W. G. *et al.* The Kondo effect in the unitary limit, Science 289, 2105–2108 (2000). 11000108 10.1126/science.289.5487.2105

[b37] Cha J. *et al.* Magnetic doping and Kondo effect in Bi_2_Se_3_ nanoribbons, Nano Lett. 10, 1076–1081 (2010). 20131918 10.1021/nl100146n

[b38] Liang C. T. *et al.* Experimental evidence for Coulomb charging effects in an open quantum dot at zero magnetic field. Phys. Rev. Lett. 81, 3507 (1998).

[b39] Amasha S. *et al.* Coulomb Blockade in an open quantum dot. Phys. Rev. Lett. 107, 216804 (2011). 22181909 10.1103/PhysRevLett.107.216804

[b40] Tkachenko O. A. *et al.* Coulomb charging effects in an open quantum dot device. J. Phys.: Condens. Matter 13, 9515 (2001).

[b41] Maurer S. M. *et al.* Coulomb blockade fluctuations in strongly coupled quantum dots. Phys. Rev. Lett. 83, 1403 (1999).

[b42] Lee J. T. *et al.* Unconventional Kondo effect in redox active single organic macrocyclic transistors, J. Am. Chem. Soc. 133, 19547–19552 (2011). 22032465 10.1021/ja208799q PMC3227745

[b43] Schmid J. *et al.* Absence of odd-even parity behavior for Kondo resonances in quantum dots, Phys. Rev. Lett. 84, 5824 (2000). 10991064 10.1103/PhysRevLett.84.5824

