# Supplementary material for: Kondo-like zero-bias conductance anomaly in a three-dimensional topological insulator nanowire
Source: Sci Rep. 2016 Feb 25;6:21767. doi: 10.1038/srep21767 (PMC4766402; doi:10.1038/srep21767)
Supplement: Supplementary Information [file srep21767-s1.pdf]

Kondo-like zero-bias conductance anomaly in a non-superconducting three-dimensional topological insulator nanowire

Sungjae Cho*[1,3], Ruidan Zhong[2], John Schneeloch[2], Genda Gu[2], Nadya Mason[§3]

[1] *Department of Physics, Department of Physics, Korea Advanced Institute of Science and Technology, Daejeon 305-701, Republic of Korea*

[2]*Condensed Matter Physics and Materials Science Department, Brookhaven National Laboratory, Upton, NY 11973, USA*

[3] *Department of Physics and Frederick Seitz Materials Research Laboratory, 104 South Goodwin Avenue, University of Illinois, Urbana, Illinois 61801, USA.*

correspondence to S. Cho(*sungjae.cho@kaist.ac.kr) or N. Mason(§nadya@illinois.edu).

Supplementary Discussion

A. Critical magnetic field of aluminum electrodes

The electrodes of our device consist of Ti(2.5nm)/Al(150nm)/Au(10nm) as described in the Method. We found the critical field value of the electrodes to be ~ 12mT by performing magneto-resistance measurement. The two-probe resistance measurement as a function of perpendicular magnetic field showed sharp transition near B=12mT as shown in Fig. S1 (blue arrows). Below this magnetic field we find that electrodes are superconducting. The measurement reported in the main manuscript was performed with magnetic field applied above this critical field value to ensure that the electrodes are non-superconducting.

B. Superconducting transport in the topological insulator nanowire device

When a magnetic field is below 12mT, the electrodes are superconducting. Therefore we observed Josephson supercurrent through the topological insulator nanowire in this magnetic field regime. Fig. S2 (a) shows differential resistance (dV/dI) as a function of gate voltages. Our nanowire device shows finite supercurrents at V_g>-50V and the critical supercurrents, the currents at the boundary between the dark blue region (dV/dI=0) and the region outside the dark blue, increase as density of states increases with the gate voltage. The critical current dependence on gate voltage is very similar to the reported Josephson effect experiment in 3D topological insulator films[1].

C. Magnetic field dependence of zero-bias conductance peaks

Fig. S3 shows two-dimensional plots of G(V_{sd},V_g) at different magnetic fields applied perpendicular to the substrate. A pair of sharp resonances crosses zero energy at Vg = -96.5V and -96.2V. These sharp resonances most likely originate from Fabry-Perot like interference. Fig. S3 shows an interesting magnetic field dependence of the zero-bias conductance anomaly. Some peaks (denoted as blue dotted-lines) do not split with magnetic field, and instead collapse to dips. The amplitudes and widths of these peaks are usually very small and we ascribe the absence of magnetic-field-induced splitting to low Kondo temperature of those peaks. Unexpectedly, we find that often conductance dips at low fields (yellow dotted-lines in Fig. S3) changes into conductance peaks as a magnetic field increases. These magnetic-field-induced peaks persist up to B=630mT. Similar phenomena were observed and explained by singlet-triplet transitions[2]. No theoretical and experimental studies about singlet-triplet transitions in topological insulator quantum dots have been reported and such phenomena are not clearly understood.

D. Two-dimensional plots of G(V_{sd},V_g) in additional gate voltage ranges

Fig. S4 shows two-dimensional plots of G(V_{sd},V_g) in the gate voltage ranges used in Fig 3 (magnetic field dependence of the peak conductance) and Fig. 4 (temperature dependence of the peak conductance) of the main article. Fig. S4 (a) and (b) show that the peak position was shifted from V_g = -72.7V(Fig. 4c) to V_g = -72.0V in a repeated gate voltage sweep due to the reconfiguration of charge traps (see the black arrow in Fig. S4 (a)). Similarly, peaks were found in a slightly shifted position in different run of 2-dimensional gate voltage and bias sweeping. The peaks observed in magnetic field dependent measurement were shifted in gate voltages from -69V(Fig. 3a) to -70.4V and -65V(Fig. 3c) to -64.2V.

Reference

1. Cho, S. et al. Symmetry protected Josephson supercurrents in three-dimensional topological insulators. *Nat. Commun.* **4**, 1689 doi: 10.1038/ncomms2701 (2013).

2. Sasaki, S. et al., Kondo effect in an integer-spin quantum dot. *Nature* **405**, 764-767 (2000).

Supplementary Figure Captions

S1| Critical magnetic field of Ti/Al/Au electrodes. (a)(b) Typical magneto-resistance of Ti/Al/Au electrodes in the device as a function of perpendicular magnetic field. **(b)** is a plot of **(a)** in a smaller range of magnetic field.

S2| superconducting transport in the topological insulator nanowire. (a) Two-dimensional plots of d*V*/d*I* versus gate voltage V_g and current I measured at B=0. The dark blue regions show the superconducting transport with zero resistance. **(b)** I-V curve cut from (a) along the yellow dotted-line shows typical supercurrent behavior.

S3| Two-dimensional plots of G(V_{sd},V_g) at different magnetic fields. Two-dimensional plots of differential conductance measured in the gate voltage ranges -96.0 V < V_g > -95.5 at four different perpendicular magnetic fields, **(a)** B=30m, **(b)** B=230m**, (c)** B=430m and **(d)** B=630m. Vertical dotted-lines denotes peak(blue) or dip(yellow) locations at zero-bias voltage at B=30mT.

S4| Additional two-dimensional plots of G(V_{sd},V_g) in the gate voltage ranges used in Fig 3 (magnetic field dependence of the peak conductance) and Fig. 4 (temperature dependence of the peak conductance) of the main article. (a) (b) two-dimensional plot of G(V_{sd},V_g) and line cut G(V_{sd},V_g = -72V) along the dotted yellow line, which covers the gate voltage range used in Fig. 4(c) (d). The black arrow in (a) shows conductance jump occuring possibly due to reconfiguration of charge traps during a gate sweep. **(c)** covers Fig. 3(c) and **(d)** covers Fig. 3(a) (b).

S1.

(a)

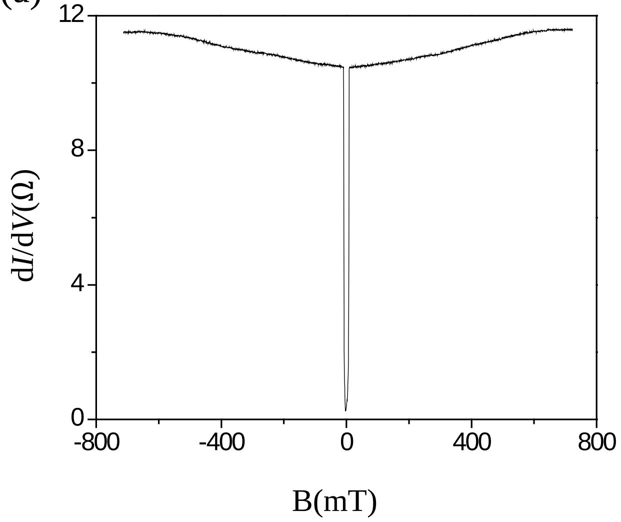

(b)

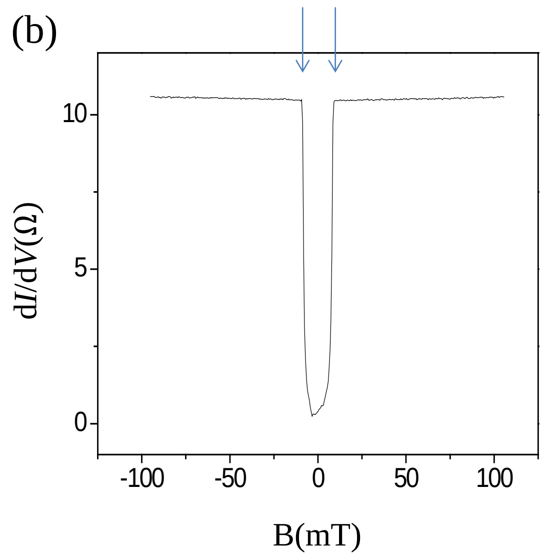

S2.

(a)

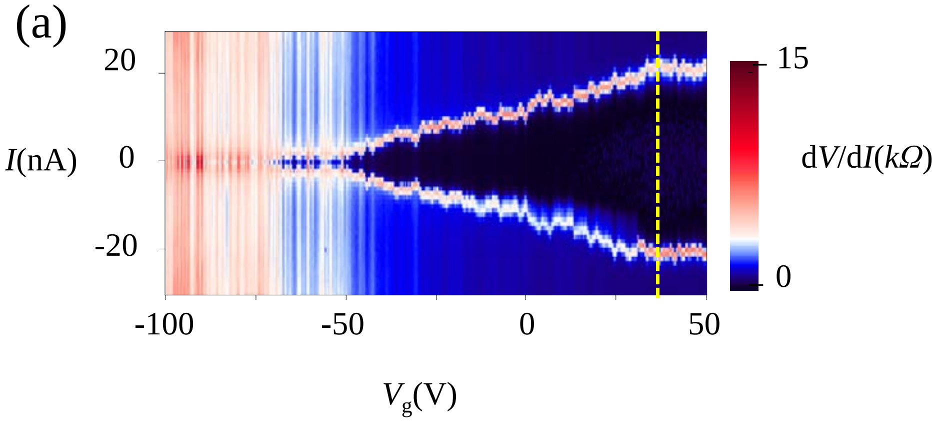

(b)

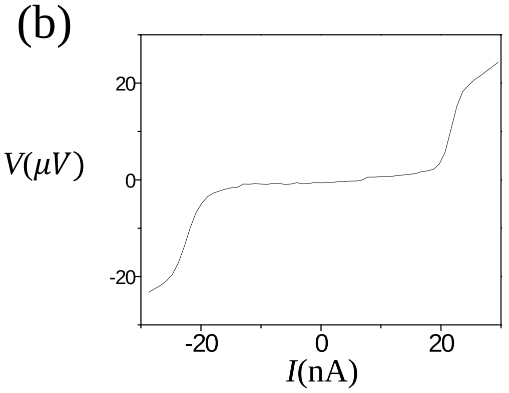

S3.

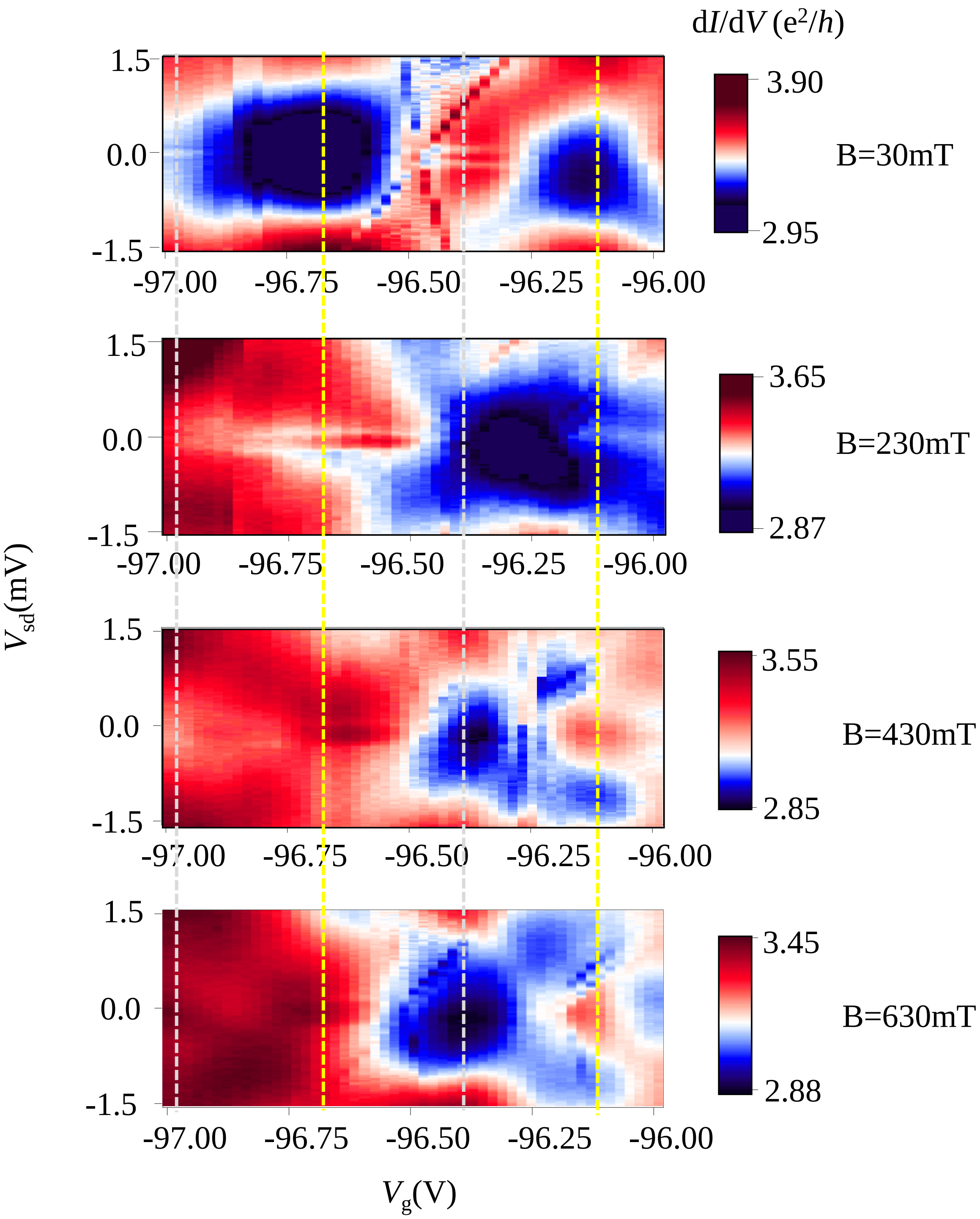

dI/dV (e²/h)
1.5
0.0
-1.5
-97.00
-96.75
-96.50
-96.25
-96.00
3.90
2.95
B=30mT
3.65
2.87
B=230mT
3.55
2.85
B=430mT
3.45
2.88
B=630mT
Vsd(mV)
Vg(V)

S4.

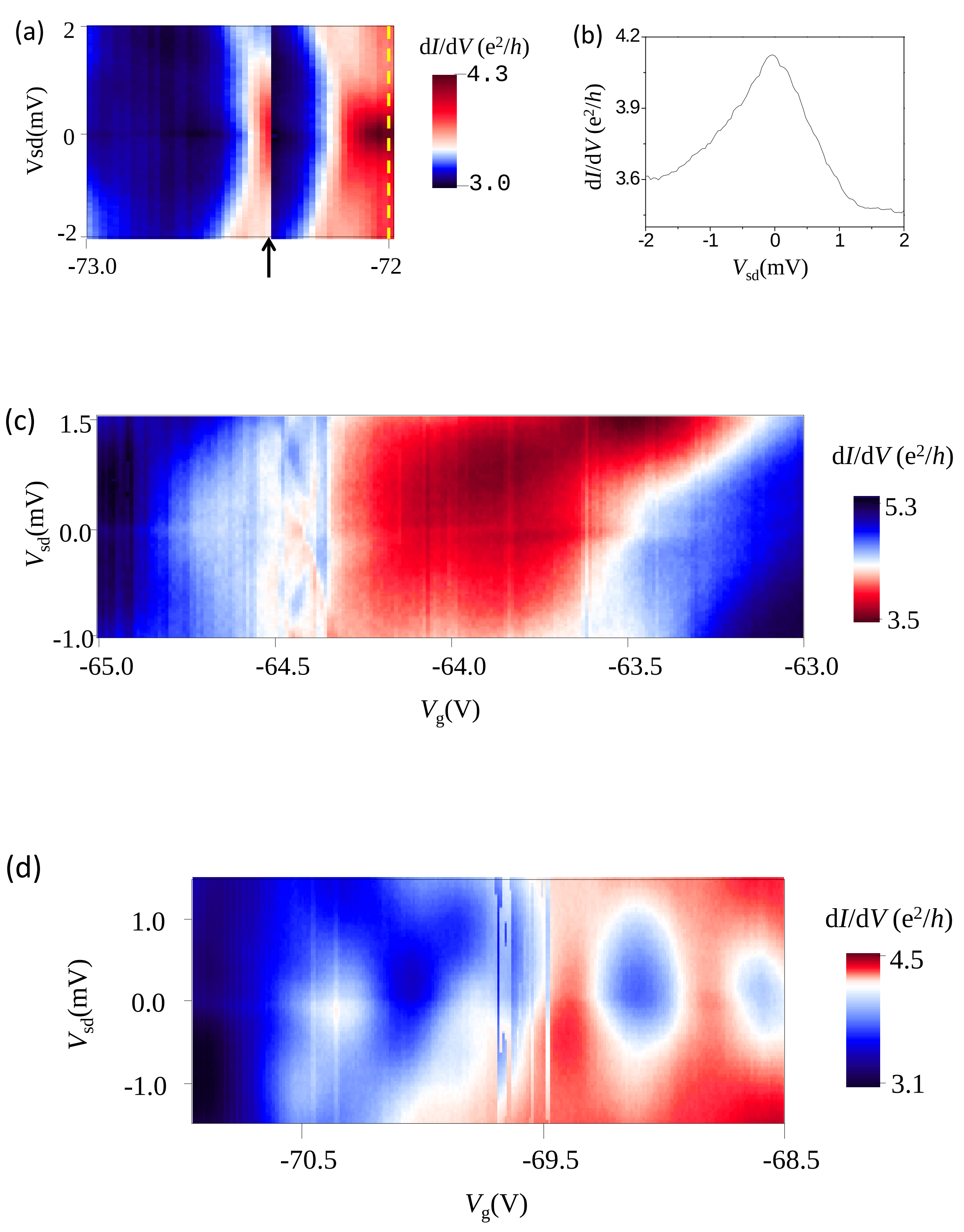
(a)
2
0
-2
Vsd(mV)
-73.0
-72
dI/dV (e2/h)
4.3
3.0
(b)
4.2
3.9
3.6
dI/dV (e2/h)
-2
-1
0
1
2
Vsd(mV)
(c)
1.5
0.0
-1.0
Vsd(mV)
-65.0
-64.5
-64.0
-63.5
-63.0
Vg(V)
dI/dV (e2/h)
5.3
3.5
(d)
1.0
0.0
-1.0
Vsd(mV)
-70.5
-69.5
-68.5
Vg(V)
dI/dV (e2/h)
4.5
3.1
